# Supplementary material for: Immunogenicity and reactogenicity of a third dose of BNT162b2 vaccine for COVID-19 after a primary regimen with BBIBP-CorV or BNT162b2 vaccines in Lima, Peru
Source: PLoS One. 2022 Oct 17;17(10):e0268419. doi: 10.1371/journal.pone.0268419 (PMC9576087; doi:10.1371/journal.pone.0268419)
Supplement: S1 Appendix — (DOCX) [file pone.0268419.s001.docx]

**S1 APPENDIX: IGG COVID-19 ANTIBODY ASSAY DESCRIPTION**

The iFlash-SARS-CoV IgG assay (Shenzen YHLO Biotech Co., Ltd, China), is a paramagnetic particle chemiluminescent immunoassay (CLIA) for semi-quantitative determination of IgG antibody to the SARS-CoV-2 spike (S protein) and nucleocapsid (N protein) in human serum or plasma using the Immunoassay Analyzer(1). This test is carried out in two important steps, the first begins with the incubation of the sample containing the anti-SARS-CoV-2 IgG, for which the pre-treatment of the sample and SARS-CoV-2 coated paramagnetic microparticles is performed, that form the antigen-antibody complex for a certain time, to proceed with the elimination of unbound materials in the solid phase of the paramagnetic field of the reaction. The second step starts by adding the acridinium-labeled antihuman IgG conjugate to be incubated again for the time required to continue with another washing process. For the reaction of the chemiluminescent signal, the pre-trigger and trigger solutions are added to the reaction mixture, which can be measured in relative light units (RLUs), the light produced by the reaction between ATP molecules and the enzyme reagent is emitted in the form of photons.

The amount of anti-SARS-CoV-2 IgG antibodies is positively correlated with the measured RLU. This method has been validated in countries like Japan and Italy showing sensitivity and specificity of 76.6% and 100% respectively for IgG (2)(3). The cut off value proposed by manufacturer is 10 AU/mL (arbitrary units per milliliter), hence all samples with IgG titers below 10 AU/mL were considered negative (non-reactive), while samples with titers more than equal to 10 AU/mL were considered positive (reactive). No lower or upper top values were specified for this assay.

1. Yokoyama R, Kurano M, Morita Y, Shimura T, Nakano Y, Qian C, et al. Validation of a new automated chemiluminescent anti-SARS-CoV-2 IgM and IgG antibody assay system detecting both N and S proteins in Japan. PLoS ONE. 2021;16(3):e0247711.
2. Infantino M, Grossi V, Lari B, Bambi R, Perri A, Manneschi M, et al. Diagnostic accuracy of an automated chemiluminescent immunoassay for anti‐SARS‐CoV‐2 IgM and IgG antibodies: an Italian experience. J Med Virol. 2020;10.1002/jmv.25932.
3. Qian C, Zhou M, Cheng F, Lin X, Gong Y, Xie et al. Development and multicenter performance evaluation of fully automated SARS-CoV-2 IgM and IgG immunoassays. Clin Chem Lab Med. 2020 Jul 15;58(9):1601-1607. doi: 10.1515/cclm-2020-0548. PMID: 32609640.
