# Supplementary material for: Immunogenicity and reactogenicity of a third dose of BNT162b2 vaccine for COVID-19 after a primary regimen with BBIBP-CorV or BNT162b2 vaccines in Lima, Peru
Source: PLoS One. 2022 Oct 17;17(10):e0268419. doi: 10.1371/journal.pone.0268419 (PMC9576087; doi:10.1371/journal.pone.0268419)
Supplement: S1 Table — (DOCX) [file pone.0268419.s002.docx]

**S1 Table:** Participant Characteristics according to Follow-up status (N=457).

|  | **Complete***  **N=285 n (%) \| Median [IQR]** | **Incomplete****  **N=172 n (%) \| Median [IQR]** | **Total**  **N=457 n (%) \| Median [IQR]** | **p-value** |
| --- | --- | --- | --- | --- |
| Age (years) | 46 [36; 60] | 42.5 [33; 60] | 45 [35; 60] | 0.081‡ |
| Age Group |  |  |  |  |
| 18-29 years old | 23 (8.1) | 16 (9.3) | 39 (8.5) | 0.846† |
| 30- 59 years old | 189 (66.3) | 110 (64.0) | 299 (65.4) |  |
| 60 plus years old | 73 (25.6) | 46 (26.7) | 119 (26.0) |  |
| Gender |  |  |  |  |
| Female | 190 (66.7) | 129 (75.0) | 319 (69.8) | 0.060† |
| Male | 95 (33.3) | 43 (25.0) | 138 (30.2) |  |
| Comorbidity |  |  |  |  |
| No Comorbidities | 214 (75.1) | 140 (81.4) | 354 (77.5) | 0.118† |
| Presence of Comorbidities | 71 (24.9) | 32 (18.6) | 103 (22.5) |  |
| List of Comorbidities |  |  |  |  |
| High Blood pressure | 29 (10.2) | 16 (9.3) | 45 (9.9) | 0.762† |
| Diabetes Mellitus | 17 (6.0) | 9 (5.2) | 26 (5.7) | 0.743† |
| Obesity | 7 (2.5) | 6 (3.5) | 13 (2.8) | 0.520† |
| Asthma/COPD | 12 (4.2) | 3 (1.7) | 15 (3.3) | 0.183†† |
| Cancer (any type) | 5 (1.8) | 0 (0.0) | 5 (1.1) | 0.162†† |
| Cardiovascular Disease | 2 (0.7) | 2 (1.2) | 4 (0.9) | 0.634†† |
| Others | 12 (4.2) | 9 (5.2) | 21 (4.6) | 0.613† |
| Prior COVID-19 Infection |  |  |  |  |
| No | 201 (70.5) | 124 (72.1) | 325 (71.1) | 0.720† |
| Yes | 84 (29.5) | 48 (27.9) | 132 (28.9) |  |
| Time until booster dose (months) |  |  |  |  |
| 5 | 33 (11.6) | 22 (12.8) | 55 (12.0) | 0.044† |
| 6 | 78 (27.4) | 67 (39.0) | 145 (31.7) |  |
| 7 | 159 (55.8) | 74 (43.0) | 233 (51.0) |  |
| 8 | 15 (5.2) | 9 (5.2) | 24 (5.3) |  |
| Vaccine Booster Regimen |  |  |  |  |
| (BNT162b2 x 2) + BNT162b2 | 56 (19.7) | 40 (23.3) | 96 (21.0) | 0.359†† |
| (BBIBP-CorV x 2) + BNT162b2 | 229 (80.3) | 132 (76.7) | 361 (79.0) |  |
| Adverse Reactions after booster |  |  |  |  |
| No | 34 (11.9) | --- | --- | --- |
| Yes | 251 (88.1) | --- | --- |  |
| Number of Adverse Reactions |  |  |  |  |
| None | 34 (11.9) | --- | --- | --- |
| One | 104 (36.5) | --- | --- |  |
| Two or more | 147 (51.6) | --- | --- |  |
| Adverse Reaction occurred |  |  |  |  |
| Local pain | 242 (84.9) | --- | --- | --- |
| Malaise | 93 (32.6) | --- | --- | --- |
| Headache | 79 (27.7) | --- | --- | --- |
| Drowsiness | 43 (15.1) | --- | --- | --- |
| Fever | 41 (14.4) | --- | --- | --- |
| Others | 54 (19.0) | --- | --- | --- |
| Time between 1st and 2nd sample (days) | 15 [14; 15] | --- | --- | --- |
| IgG Titers (AU/ml) |  |  |  |  |
| Before booster | 29.1 [8.4; 93.1] | 27.3 [7.2; 96.0] | 28.3 [8.3; 96.0] | 0.718‡ |
| After booster | 501.9 [446.8; 545.4] | --- | --- | --- |
| Natural Logarithms of IgG titers |  |  |  |  |
| Before booster | 3.4 [2.1; 4.5] | 3.3 [2.0; 4.6] | 3.3 [2.1; 4.6] | 0.718‡ |
| After booster | 6.2 [6.1; 6.3] | --- | --- | --- |
| IQR: Interquartile range. IgG: Immunoglobulin G. AU/ml: Arbitrary units per ml. | | | | |
| * Complete: participant had two blood samples measures: 1) On the same day of the vaccine booster but before receiving the dose, 2) Post vaccine booster but no later than 28 days after the dose.  ** Incomplete: Participants either had only the initial measurement, or the second measurement was done more than 28 days after vaccine booster dose.  †Chi Square test. ††Fisher´s Exact test. ‡Mann-Whitney U test. | | | | |
