# Supplementary material for: Immunogenicity and reactogenicity of a third dose of BNT162b2 vaccine for COVID-19 after a primary regimen with BBIBP-CorV or BNT162b2 vaccines in Lima, Peru
Source: PLoS One. 2022 Oct 17;17(10):e0268419. doi: 10.1371/journal.pone.0268419 (PMC9576087; doi:10.1371/journal.pone.0268419)
Supplement: S2 Table — (DOCX) [file pone.0268419.s003.docx]

**S2 Table:** Characteristics of Participants with complete Follow-up according to presence of adverse reactions to the vaccine booster (N=285).

|  | **No Adverse Reaction**  **N=34 n (%) \| Median [IQR]** | **Adverse Reactions**  **N=251 n (%) \| Median [IQR]** | **Total**  **N=285 n (%) \| Median [IQR]** | **p value** |
| --- | --- | --- | --- | --- |
| Age (years) | 58.5 [38; 69] | 45 [35; 59] | 46 [36; 60] | 0.008‡ |
| Age Group |  |  |  |  |
| 18-29 years old | 0 (0.0) | 23 (100.0) | 23 | 0.012† |
| 30- 59 years old | 19 (10.1) | 170 (89.9) | 189 |  |
| 60 plus years old | 15 (20.6) | 58 (79.4) | 73 |  |
| Gender |  |  |  |  |
| Female | 15 (7.9) | 175 (92.1) | 190 | 0.003† |
| Male | 19 (20.0) | 76 (80.0) | 95 |  |
| Comorbidity |  |  |  |  |
| No Comorbidities | 22 (10.3) | 192 (89.7) | 214 | 0.136† |
| Presence of Comorbidities | 12 (16.9) | 59 (83.1) | 71 |  |
| Number of Comorbidities |  |  |  |  |
| No Comorbidities | 22 (10.3) | 192 (89.7) | 214 | 0.253† |
| One Comorbidity | 11 (18.0) | 50 (82.0) | 61 |  |
| Two or more Comorbidities | 1 (10.0) | 9 (90.0) | 10 |  |
| Prior COVID-19 Infection |  |  |  |  |
| No | 28 (13.9) | 173 (86.1) | 201 | 0.107† |
| Yes | 6 (7.1) | 78 (92.9) | 84 |  |
| Time until booster dose (months) |  |  |  |  |
| 5 | 2 (6.1) | 31 (93.9) | 33 | 0.065†† |
| 6 | 16 (20.5) | 62 (79.5) | 78 |  |
| 7 | 15 (9.4) | 144 (90.6) | 159 |  |
| 8 | 1 (6.7) | 14 (93.3) | 15 |  |
| Vaccine Booster Regimen |  |  |  |  |
| (BNT162b2 x 2) + BNT162b2 | 13 (23.2) | 43 (76.8) | 56 | 0.004†† |
| (BBIBP-CorV x 2) + BNT162b2 | 21 (9.2) | 208 (90.8) | 229 |  |
| IgG Titers before booster (AU/ml) | 18.9 [8.1; 93.1] | 30.7 [8.6; 98.6] | 29.1 [8.4; 93.1] | 0.602‡ |
| Natural Log of IgG titers before booster | 2.9 [2.1; 4.5] | 3.4 [2.1; 4.6] | 3.4 [2.1; 4.5] | 0.602‡ |
| IQR: Interquartile range. IgG: Immunoglobulin G. AU/ml: Arbitrary units per ml. | | | | |
| †Chi Square test. ††Exact test. ‡Mann-Whitney U test. | | | | |
