# Supplementary material for: Immunogenicity and reactogenicity of a third dose of BNT162b2 vaccine for COVID-19 after a primary regimen with BBIBP-CorV or BNT162b2 vaccines in Lima, Peru
Source: PLoS One. 2022 Oct 17;17(10):e0268419. doi: 10.1371/journal.pone.0268419 (PMC9576087; doi:10.1371/journal.pone.0268419)
Supplement: S3 Table — (DOCX) [file pone.0268419.s004.docx]

**S3 Table:** IgG Geometric Mean Titers (AU/ml) before and after receiving the COVID-19 vaccine booster dose stratified by Booster Regimen (N=285).

|  | **(BNT162b2 x 2) + BNT162b2** **Booster Regimen** | | | | |  | **(BBIBP-CorV x 2) + BNT162b2 Booster Regimen** | | | | |
| --- | --- | --- | --- | --- | --- | --- | --- | --- | --- | --- | --- |
|  | **Baseline Geometric Mean**  **(GSD)** | **p-value*** |  | **After booster Geometric Mean**  **(GSD)** | **p-value*** |  | **Baseline Geometric Mean**  **(GSD)** | **p-value*** |  | **After booster Geometric Mean**  **(GSD)** | **p-value*** |
| **Age Group** |  |  |  |  |  |  |  |  |  |  |  |
| 18-29 years old | --- |  |  | --- |  |  | 22.9 (5.4) | 0.834 |  | 518.9 (1.1) | 0.076 |
| 30-59 years old | 100.9 (2.0) | 0.977 |  | 452.1 (1.2) | 0.248 |  | 20.4 (4.5) |  |  | 507.2 (1.1) |  |
| 60 plus years old | 99.4 (3.3) |  |  | 411.9 (1.2) |  |  | 24.5 (6.9) |  |  | 480.2 (1.2) |  |
| **Gender** |  |  |  |  |  |  |  |  |  |  |  |
| Female | 83 (3.4) | 0.237 |  | 418.4 (1.2) | 0.821 |  | 21.4 (4.7) | 0.802 |  | 502.1 (1.1) | 0.178 |
| Male | 119.4 (2.9) |  |  | 413.6 (1.2) |  |  | 20.2 (5.0) |  |  | 514.1 (1.1) |  |
| **Comorbidity** |  |  |  |  |  |  |  |  |  |  |  |
| No Comorbidities | 92.8 (2.7) | 0.652 |  | 427 (1.2) | 0.296 |  | 23.1 (4.7) | 0.063 |  | 509.6 (1.1) | 0.067 |
| Presence of Comorbidities | 106.7 (3.6) |  |  | 405.2 (1.2) |  |  | 13.9 (4.9) |  |  | 488.5 (1.1) |  |
| **Number of Comorbidities** |  |  |  |  |  |  |  |  |  |  |  |
| No comorbidities | 92.8 (2.7) | 0.674 |  | 427 (1.2) | 0.578 |  | 23.1 (4.7) | 0.156 |  | 509.6 (1.1) | 0.128 |
| One comorbidity | 97.8 (3.9) |  |  | 406 (1.2) |  |  | 14.2 (4.5) |  |  | 488.2 (1.1) |  |
| Two or more comorbidities | 147.2 (2.8) |  |  | 402.6 (1.2) |  |  | 11.7 (12.5) |  |  | 491.8 (1.2) |  |
| **Prior COVID-19 infection** |  |  |  |  |  |  |  |  |  |  |  |
| No Infection | 76.9 (2.9) | <0.001 |  | 400.5 (1.2) | <0.001 |  | 13.6 (4.5) | <0.001 |  | 497 (1.1) | 0.003 |
| Prior Infection | 285.4 (2.1) |  |  | 486.1 (1.1) |  |  | 53 (3.6) |  |  | 524.3 (1.1) |  |
| **Time until booster dose (months)** |  |  |  |  |  |  |  |  |  |  |  |
| 5 | 112.6 (2.9) | 0.416 |  | 424.3 (1.2) | 0.087 |  | --- |  |  | --- |  |
| 6 | 76.6 (3.8) |  |  | 392.5 (1.2) |  |  | 21.4 (4.5) | 0.041 |  | 490.8 (1.2) | 0.081 |
| 7 | 147.2 (1.3) |  |  | 492.7 (1.2) |  |  | 19 (4.7) |  |  | 511.7 (1.1) |  |
| 8 | --- |  |  | --- |  |  | 55.2 (6.5) |  |  | 499.7 (1.1) |  |
| IgG: Immunoglobulin G. AU/ml: Arbitrary units per ml. GSD: geometric standard deviation.  * Student T or F test for geometric means of each category. | | | | | |  |  |  |  |  |  |
