# Supplementary material for: Immunogenicity and reactogenicity of a third dose of BNT162b2 vaccine for COVID-19 after a primary regimen with BBIBP-CorV or BNT162b2 vaccines in Lima, Peru
Source: PLoS One. 2022 Oct 17;17(10):e0268419. doi: 10.1371/journal.pone.0268419 (PMC9576087; doi:10.1371/journal.pone.0268419)
Supplement: S4 Table — (DOCX) [file pone.0268419.s005.docx]

**S4 Table:** IgG Median Titers (AU/ml) before (baseline) and after receiving the COVID-19 vaccine booster dose (N=285).

|  | **Baseline Median**  **(Percentile 25th; 75th)** | **p-value*** |  | **After Booster Median**  **(Percentile 25th; 75th)** | **p-value*** |
| --- | --- | --- | --- | --- | --- |
| Age Group |  |  |  |  |  |
| 18-29 years old | 20.3 (8.7; 71.4) | <0.001 |  | 535.9 (481.2; 555.6) | <0.001 |
| 30-59 years old | 18.0 (7.4; 71.7) |  |  | 514.2 (471.6; 551.4) |  |
| 60 plus years old | 77.4 (32.1; 219.0) |  |  | 438.7 (389.5; 498.9) |  |
| Gender |  |  |  |  |  |
| Female | 24.6 (8.4; 82) | 0.199 |  | 498.4 (447.9; 546.3) | 0.821 |
| Male | 41.7 (8.6; 123.3) |  |  | 505.3 (448.1; 539.8) |  |
| Comorbidity |  |  |  |  |  |
| No Comorbidities | 24.8 (8.3; 90.7) | 0.435 |  | 511.5 (468.1; 550.3) | <0.001 |
| Presence of Comorbidities | 40.9 (10.3; 99.3) |  |  | 467.7 (420.3; 522.3) |  |
| Number of Comorbidities |  |  |  |  |  |
| No comorbidities | 24.8 (8.3; 90.7) | 0.248 |  | 511.5 (468.1; 550.3) | <0.001 |
| One comorbidities | 34.2 (8.5; 90.7) |  |  | 467.7 (422.7; 522.5) |  |
| Two or more comorbidities | 80.1 (33.3; 225.7) |  |  | 451.1 (371.8; 510.6) |  |
| Prior COVID-19 infection |  |  |  |  |  |
| No Infection | 16.8 (7.0; 67.1) | <0.001 |  | 489.9 (434.5; 530.2) | <0.001 |
| Prior Infection | 78.2 (26.4; 164.9) |  |  | 540.3 (479.3; 560.1) |  |
| Time until booster dose (months) | |  |  |  |  |
| 5 | 101.2 (54.6; 315.8) | <0.001 |  | 432.6 (395.9; 476.4) | <0.001 |
| 6 | 33.5 (8.2; 92.5) |  |  | 474.6 (419.3; 532.3) |  |
| 7 | 16.0 (7.1; 68.1) |  |  | 523.2 (477.6; 554.2) |  |
| 8 | 78.9 (17.9; 277.9) |  |  | 489.3 (476.9; 537.3) |  |
| Vaccine Booster Regimen |  |  |  |  |  |
| (BNT162b2 x 2) + BNT162b2 | 101.9 (53.7; 216.9) | <0.001 |  | 419.7 (368.9; 472.8) | <0.001 |
| (BBIBP-CorV x 2) + BNT162b2 | 17.4 (7.3; 70.9) |  |  | 517.1 (472.6; 552.2) |  |
| IgG: Immunoglobulin G. AU/ml: Arbitrary units per ml. GSD: geometric standard deviation.  * Mann-Whitney or Kruskall-Wallis test. | | | | | |
