# Supplementary material for: Immunogenicity and reactogenicity of a third dose of BNT162b2 vaccine for COVID-19 after a primary regimen with BBIBP-CorV or BNT162b2 vaccines in Lima, Peru
Source: PLoS One. 2022 Oct 17;17(10):e0268419. doi: 10.1371/journal.pone.0268419 (PMC9576087; doi:10.1371/journal.pone.0268419)
Supplement: S5 Table — (DOCX) [file pone.0268419.s006.docx]

**S5 Table:** Adjusted Quantile Regression Model using IgG levels (AU/ml) after vaccine booster as outcome showing coefficients for each spline (N=285).

|  | **IgG Titers after Booster (AU/ml)** | |
| --- | --- | --- |
|  | **MD (95% CI)** | **p value ^a^** |
| **Age (years) ^b^** |  |  |
| Spline1 (<36) | -0.57 (-5.22; 4.08) | 0.809 |
| Spline2 (36-46) | 14.46 (-27.48; 56.40) | 0.498 |
| Spline3 (46-59) | -46.20 (-144.8; 52.42) | 0.357 |
| Spline4 (>59) | 57.99 (-31.53; 147.5) | 0.203 |
| **Gender** |  |  |
| Female | Reference |  |
| Male | 5.39 (-7.68; 18.46) | 0.417 |
| **Comorbidity** |  |  |
| No Comorbidities | Reference |  |
| Presence of Comorbidities | 1.88 (-14.24; 17.99) | 0.819 |
| **Prior COVID-19 Infection** |  |  |
| No | Reference |  |
| Yes | 29.11 (11.49; 46.73) | 0.001 |
| **Time until booster dose (days) ^b^** |  |  |
| Spline1 (<204) | -0.55 (-2.69; 1.59) | 0.610 |
| Spline2 (204-222) | 0.19 (-3.18; 3.55) | 0.913 |
| Spline3 (222-234) | 10.65 (-22.23; 43.5) | 0.524 |
| Spline4 (>234) | -72.36 (-191.5; 46.8) | 0.233 |
| **Vaccine Booster Regimen** |  |  |
| BNT162b2 + BNT162b2 | Reference |  |
| BBIBP-CorV + BBIBP-CorV | 92.3 (24.90; 159.7) | 0.007 |
| **Time between 1st and 2nd sample ^b^** |  |  |
| Spline1 (13-15) | 9.73 (-4.57; 24.03) | 0.182 |
| Spline2 (15-28) | -15.83 (-36.18; 4.52) | 0.127 |
| **Natural Logarithm of IgG titers before Booster ^b^** |  |  |
| Spline1 (<0.99) | 71.79 (23.49; 120.1) | 0.004 |
| Spline2 (0.99-1.46) | -222.8 (-456.2; 10.65) | 0.061 |
| Spline3 (1.46-1.94) | 716.0 (-489.1; 1921.1) | 0.243 |
| Spline4 (>1.94) | -352.3 (-2432.2; 1727.6) | 0.739 |
| MD: Adjusted Median Difference. 95%CI: 95% Confidence Interval.  a All p-values was obtained using a robust standard error estimator to address heteroskedasticity.  b The non-linear effect of age, time until booster dose, time between 1st and 2nd sample and natural log of IgG titers before booster in multivariable linear regression are shown in **Figure 4**. | | |
