# Supplementary material for: Immunogenicity and reactogenicity of a third dose of BNT162b2 vaccine for COVID-19 after a primary regimen with BBIBP-CorV or BNT162b2 vaccines in Lima, Peru
Source: PLoS One. 2022 Oct 17;17(10):e0268419. doi: 10.1371/journal.pone.0268419 (PMC9576087; doi:10.1371/journal.pone.0268419)
Supplement: S6 Table — (DOCX) [file pone.0268419.s007.docx]

**S6 Table:** Adjusted Linear Regression Model using IgG levels (AU/ml) after vaccine booster as outcome showing coefficients for each spline (N=285).

|  | **IgG Titers after Booster (AU/ml)** | |
| --- | --- | --- |
|  | **GMR (95% CI)** | **p value ^a^** |
| **Age (years) ^b^** |  |  |
| Spline1 (<46) | 0.95 (0.85; 1.07) | 0.417 |
| Spline2 (>46) | 0.88 (0.7; 1.11) | 0.272 |
| **Gender** |  |  |
| Female | Reference |  |
| Male | 1.01 (0.97; 1.04) | 0.650 |
| **Comorbidity** |  |  |
| No Comorbidities | Reference |  |
| Presence of Comorbidities | 0.98 (0.94; 1.03) | 0.460 |
| **Prior COVID-19 Infection** |  |  |
| No | Reference |  |
| Yes | 1.06 (1.02; 1.1) | 0.004 |
| **Time until booster dose (days) ^b^** |  |  |
| Spline1 (<205.2) | 1.06 (0.91; 1.24) | 0.448 |
| Spline2 (205-234) | 0.8 (0.52; 1.22) | 0.297 |
| Spline3 (>234) | 0.95 (0.82; 1.09) | 0.456 |
| **Vaccine Booster Regimen** |  |  |
| BNT162b2 + BNT162b2 | Reference |  |
| BBIBP-CorV + BBIBP-CorV | 1.13 (1.01; 1.27) | 0.041 |
| **Time between 1st and 2nd sample ^b^** |  |  |
| Spline1 (<14) | 0.99 (0.9; 1.09) | 0.827 |
| Spline2 (14-15) | 1.35 (0.96; 1.91) | 0.089 |
| Spline32 (>15) | 1.02 (0.81; 1.27) | 0.896 |
| **Natural Logarithm of IgG titers before Booster ^b^** |  |  |
| Spline1 (<1.12) | 71.79 (23.49; 120.1) | 0.004 |
| Spline2 (1.12-1.82) | -222.8 (-456.2; 10.65) | 0.061 |
| Spline3 (>1.82) | -352.3 (-2432.2; 1727.6) | 0.739 |
| GMR: Adjusted Geometric Mean Ratio. 95%CI: 95% Confidence Interval.  a All p-values was obtained using a robust standard error estimator to address heteroskedasticity.  b The non-linear effect of age, time until booster dose, time between 1st and 2nd sample and natural log of IgG titers before booster in multivariable linear regression are shown in **Figure 4**. | | |
