# Supplementary material for: Immunogenicity and reactogenicity of a third dose of BNT162b2 vaccine for COVID-19 after a primary regimen with BBIBP-CorV or BNT162b2 vaccines in Lima, Peru
Source: PLoS One. 2022 Oct 17;17(10):e0268419. doi: 10.1371/journal.pone.0268419 (PMC9576087; doi:10.1371/journal.pone.0268419)
Supplement: Attachment — Submitted filename: Response to Reviewers.docx [file pone.0268419.s009.docx]

Manuscript reference number: PONE-D-22-12508

Title: Immunogenicity and reactogenicity of a third dose of BNT162b2 vaccine for COVID-19 after a primary regimen with BBIBP-CorV or BNT162b2 vaccines in Lima, Peru.

Dear Emily Chenette

Senior Editor,

Plos One

We thank the editor and the two reviewers for their comments on our manuscript, below is our response to each point. We are uploading a version of the manuscript with track changes, and a clean version with all the changes accepted. We hope that we satisfyingly addressed all the comments and that the manuscript will be now suited for publication

Best Regards,

On behalf of all authors

Natalia Vargas, MD

Instituto Nacional de Salud (INS)

Lima, Peru

August 16^th^ 2022

AUTHOR´S RESPONSE TO COMMENTS:

Reviewer #1:

- *IgG levels are determined using an assay that tests for reactivity against both the spike protein and the nucleoprotein of SARS-CoV-2 without differentiating this reactivity. Because the BBIBP-CorV vaccine is an inactivated whole virus vaccine, it will induce antibodies against both proteins (spike and nucleoprotein). In contrast, the BNT162b2 mRNA vaccine exclusively induces antibodies against the spike. This complicates comparisons of the IgG levels as the assay used will pick up two classes of antibodies after BBIBP-CorV vaccination but only class after BNT162b2 vaccination. Ideally, samples would be analysed using an IgG assay that determines only spike reactivity. Alternatively, could the contribution of anti-N antibodies to the determined signal be analysed for a representative subset of samples? At a minimum, the use of a dual-reactive assay should be discussed as a limitation for the interpretation of this study.*

**RE:** Thank you for your comment, we have added that limitation in lines 381-385 as suggested.

- *Immunogenicity results are provided as AU/ml (arbitrary units/ml). To facilitate comparison to results of other trials, it would be helpful if a conversion factor to the international standard BAU/ml using the WHO reference sample with this assay could be provided.*

**RE:** We truly appreciate your recommendation. Unfortunately, due to logistical restrictions we were able to access this test which does not have yet a BAU conversion factor. Nevertheless, the ratios and relationships found in AU/ml with this test correlate with BAU/ml

- *Lines 224-226: Numbers in the text (RR 1.13, 95% CI 1.02-1.26) do not match numbers in Table 2 (female gender, adjusted model; RR 1.12, 95% CI 1.01-1.25)*

**RE:** Thank you for this observation, this was a typo. We have proceeded to correct the numbers in the text (lines 225-226) according to the information provided in Table 2.

- *Lines 243-245/Table 3: The higher baseline titers in older individuals are not unexpected as the majority of elderly individuals had received primary immunizations with the much more immunogenic BNT162b2 vaccine. This should be pointed out here. Because the BNT162b2 is overall more immunogenic than the BBIBP-CorV vaccine, it would be helpful to have Table 3 (or an additional table) be split up into the different primary vaccines as well.*

**RE:** Thank you for pointing this out. We have added this information in lines 244-246 as “A possible explanation was that elderly were immunized with the BNT162b2 vaccine which has demonstrated to be more immunogenic than BBIBP-CorV according to some studies”.

- *Lines 338-342: While protection induced by inactivated virions could potentially be wider, the statement in its current form is a bit misleading as the lower protection of inactivated virion vaccines compared to the highly immunogenic mRNA vaccines seems well established.*

RE: Thank you for your comment, we have proceeded to clarify this concept in line 345 as “In the particular case of inactivated COVID-19 vaccines such as BBIBP-CorV, as these contain additional SARS-CoV-2 proteins such as the nucleoprotein, could produce a wider immunological response than produced by the spike protein”. We were referring to the immune response and not to the protection of the BBIBP-CorV vaccine; we have also specified that it is a theoretical possibility rather than an established fact.

- *Lines 349-351: The authors state that antibody titers before the booster inversely correlated with the post-boost titers, suggesting that shorter boosting intervals may not be beneficial. In this study, this observation is very likely to be confounded by the fact that young individuals, which generally respond to the BNT162b2 vaccine with higher IgG titers than older individuals, had received the much less immunogenic BBIBP-CorV vaccine first. This results in the relatively strong increase in younger individuals (poorly immunogenic first vaccine, very strong response to booster). In contrast, older individuals had higher baseline titers due to the more immunogenic BNT162b2 vaccine compared to BBIBP-CorV (although titers in younger individuals receiving BNT162b2 would have been even higher), and then responded less strongly to the BNT162b2 booster dose than younger individuals.*

**RE:** Thank you for your comment, we have deleted the sentence about short interval boosting, in line 354. We left the statement just as an observation that antibody titers before the booster inversely correlated with the titters after booster with an mRNA vaccine, as stated by Goel et al.

- *Table 1: While i.) the elapsed time between the second vaccine dose and the booster dose as well as ii.) the time between the booster dose and the follow-up visit are described, information of the time between the first and second dose is missing (line 86 says: at least 21 days apart). As prolonged vaccination intervals are known to affect the humoral vaccine response, it would be helpful for the interpretation of the results and comparison of the groups to also include this measure.*

**RE:** Thank you for your comment. We calculated the median time between the first and second dose, but we left those results out of the manuscript to keep the text length under the number of words required. The time between first and second doses ranged between 20 and 68 days. The median time between first and second doses was 21 days (IQR: 21 - 21). We are including this information in lines 86 and 87.

- *Table 1: Make clear within the table what groups the p values are comparing.*

**RE:** P values are result of comparing the (BNT162b2 x 3) group versus (BBIBP-CorV x 2 + BNT162b2) group. We have edited Table 1 to clarify the interpretation of the p values.

*Figure 2: Instead of the natural logarithm, it appears advisable to display the data in the much more commonly used log10 format, which will help with comparison to other results. In addition, the legend needs to define lines, boxes and whiskers.*

**RE:** Thank you for this suggestion. There is a big change in the use of log10 or ln, for example references(1)(2)use log10, while(3) use ln. There seems to be no consensus or pre-established guideline on which one should be used over the other; however, from a statistical point of view, the results should not differ beyond what is explained by numerical accuracy. We have performed this checking on the data, for instance we have considered to use the natural logarithm.

- *Figure 3: The 95% confidence intervals are a bit difficult to make out and it would be helpful to display them in a more prominent shade of grey.*

**RE:** Thank you for your comment. We have proceeded to increase the intensity of the grey shading to improve visibility.

- *Line 65: “13% fold” does not make sense*

**RE:** Thank you for the comment, we have proceeded to delete the word “fold” in lines 65 and 285.

- *Line 67: Make clear what “both” is referring to.*

**RE:** Thank you for the comment and we have clarified in line 67 that “both” refers to the two vaccine regimens (heterologous and homologous) assessed along this study.

- *Line 144: Typo, should be BNT162b2.*

**RE:** Thank you for the comment and we apologise for the typo. We have proceeded to correct to BNT162b2 in line 144.

- *Line 201: Ranged would be a better word than oscillated.*

**RE:** Thank you for the comment, we have proceeded to put “oscillated” instead of “ranged” in line 201.

- *Lines 212-213: Better to write that there were “no statistically significant” differences than just “no” differences.*

**RE:** Thank you for the comment and we have proceeded to add “no statistically significant” in line 212.

- *Line 221: Better: nausea than nauseas.*

**RE:** Thank you for the comment, we have proceeded to correct the word as suggested in line 221.

- *Line 309: The lower antibody levels could also be expected because of the overall lower immunogenicity of the BBIBP-CorV vaccine compared to BNT162b2.*

**RE:** Thank you for the comment, we have proceeded to add this sentence in lines 311 and 312, and a reference.

- *Line 331-332: Heterologous vaccines are common ‘practice’ for several of the diseases (e.g., HIV, HPV). ‘Has been investigated’ might therefore be a better choice of words than ‘has been practiced’.*

**RE:** Thank you for the comment, we have proceeded to replace the word “practiced” with “investigated” in line 335.

- *Line 376: While the authors rightfully acknowledge the lack of analyses on neutralizing serum titers as a limitation of the study, the term ‘broadly’ for the description of the extend of humoral response measurements seems too wide to me (it is a rather narrow measurement of the humoral response). One could rather write “overall binding reactivity” or something similar.*

**RE:** Thank you for the comment. We have proceeded to add the phrase “by the overall binding reactivity” in line 388.

- *Line 395: “In addition, the antibody titers rising trend after the second vaccine dose in our study indicates that subsequent boosters could be spaced and prioritized in certain populations such as elderly and immunosuppressed.” I am not entirely sure what this statement is referring to. Trends after the second vaccine dose (prior to the booster) were not investigated. Should this refer to the higher titers after the booster dose compared to after the second vaccine dose?*

**RE:** Thank you for the comment and we apologise for this typo. We meant to refer to the third dose, not the second dose. We have proceeded to make the correction in line 407.

Reviewer #2:

*- Overall, the manuscript is written with a reasonable standard of English but could do with a thorough grammar edit.*

*The study is interesting, but it is difficult to draw any major conclusions about the impact of booster vaccines for the following reasons:*

**RE:** Thank you for your comments, we have revised the English grammar along the manuscript.

*- It is expected that there would be such an increase in antibody titres early after a booster dose, 2 weeks is too early to assess immunogenicity and durability of response and a later sample would be more meaningful in response interpretation.*

**RE:** In this study we aimed to evaluate immediate immunogenicity and observed the antibody peak. However, we agree with your comment, that is the reason we are following these patients for the next six months in order to assess long term immunogenicity and durability of antibody response.

*- Only IgG was measured, not neutralising antibody which may be more accurate in interpreting immune response correlating with protection*

**RE:** Thank you for this comment, in the limitations section of the article in lines 388 and 389, we have mentioned that we did not include neutralizing antibodies response.

*- In view of this, is preferable to tone these findings down as unfortunately it may be misleading to say that these responses result in protection and to correlate them to protection. The findings are interesting, but need to be focussed on what can be derived, rather than making assumptions*

**Re:** We agree with you that the word “protection” is misleading, so we have proceeded to clarify this concept along the text. In the discussion, we tried to convene the idea that inactivated virions could theoretically produce a wider set of antibodies, not that they could produce a better response or protection. Therefore, we have modified several lines toning down phrases regarding protection and possible vaccine effectiveness, focusing just on immunogenicity. *-There are also substantial differences between the homologous and heterologous groups and a comparison is very difficult even with some parameters controlled for*

**RE:** Thank you for your comment. We are aware that the homologous and heterologous groups have pronounced differences, particularly the median age of participants, and the time between boosting and second dose. However, this is the best available data to observe immunogenic response to boosting between groups that were primed differently. Differences in age and time to boosting come from the way the vaccine program rolled up in Peru, and therefore it was almost impossible to have matching comparable groups. In order to control this, we used the best adjustment strategies for observational studies; however, we recognized that residual confounding is a real possibility. We have added this limitation in lines 385-388 as “another important limitation is that due to the vaccine program rolled up in Peru, there were pronounced differences between participants characteristics vaccinated with the homologous and the heterologous regimen, particularly the median age, however, we used the best adjustment strategies for observational studies”.

In addition, all observational studies have an inherent risk of confounding which can be reduced by regression adjustment strategies. Our study adjusted for known confounding variables such as age, gender, comorbidities, prior COVID-19 infection, time until booster, time between samples and baseline IgG levels, this is mentioned in lines 327 and 328. However, the risk of residual confounding will always be present in any observational study.

- *In addition:*

*- How was fever assessed?*

**RE:** Thank you for the comment. We assessed fever by asking participants if they had an oral temperature of 38°C or above. We have added to the manuscript (lines 143 and 144) that safety assessment was self-reported and adverse reactions were inquired during the two-week follow-up visit

*-Was any formal measurement of local reactogenicity conducted (measure size of swelling, erythema etc.) Was FDA guidance used to evaluate this?*

**RE:** Reactogenicity was self-reported, as mentioned in lines 143 and 144. We used the Common Terminology Criteria for Adverse Events (CTCAE) as guidance to evaluate reactogenicity.

*- How was prior COVID-19 assessed, given that it is expected that by Dec 21 close to 80% of the global population had been infected with COVID-19 as evidenced by antibody testing. Even asymptomatic infection would give an Ab response and this is likely to confound baseline data.*

**RE:** We assessed prior COVID-19 and/or documented SARS-CoV-2 infection using a composite definition based on self-reporting and having a prior positive antigenic or molecular test available in the Integrated COVID-19 Register of antigenic and molecular tests (SISCOVID) from the Ministry of health. Asymptomatic infections with no registry of diagnostic tests were not accounted in the study. We are adding this as a limitation: “A further limitation is the under-reporting of prior SARS-CoV-2 due to presence of asymptomatic infections with no testing” in lines 380 – 382.

*- In line 225 heterologous has been duplicated- I'm assuming that homologous is the correct term.*

*RE:* Thank you for the comment, “homologous” is the correct term, and it was edited accordingly in the line 277.

REFERENCES

1.	Ligumsky H, Safadi E, Etan T, Vaknin N, Waller M, Croll A, et al. Immunogenicity and Safety of the BNT162b2 mRNA COVID-19 Vaccine Among Actively Treated Cancer Patients. JNCI J Natl Cancer Inst. 1 de febrero de 2022;114(2):203-9.

2.	Pollock KM, Cheeseman HM, Szubert AJ, Libri V, Boffito M, Owen D, et al. Safety and immunogenicity of a self-amplifying RNA vaccine against COVID-19: COVAC1, a phase I, dose-ranging trial. eClinicalMedicine. 1 de febrero de 2022;44:101262.

3.	Haranaka M, Baber J, Ogama Y, Yamaji M, Aizawa M, Kogawara O, et al. A randomized study to evaluate safety and immunogenicity of the BNT162b2 COVID-19 vaccine in healthy Japanese adults. Nat Commun. 14 de diciembre de 2021;12(1):7105.
