# Supplementary material for: Immunogenicity and reactogenicity of a third dose of BNT162b2 vaccine for COVID-19 after a primary regimen with BBIBP-CorV or BNT162b2 vaccines in Lima, Peru
Source: PLoS One. 2022 Oct 17;17(10):e0268419. doi: 10.1371/journal.pone.0268419 (PMC9576087; doi:10.1371/journal.pone.0268419)
Supplement: Attachment — Submitted filename: Response to Reviewers.docx [file pone.0268419.s010.docx]

Manuscript reference number: PONE-D-22-12508R1

Title: Immunogenicity and reactogenicity of a third dose of BNT162b2 vaccine for COVID-19 after a primary regimen with BBIBP-CorV or BNT162b2 vaccines in Lima, Peru.

Dear Emily Chenette

Senior Editor,

Plos One

We thank the editor and the reviewer #1 for this new round of comments on our manuscript, below is our response to each point. We are uploading a version of the manuscript with track changes, and a clean version with all the changes accepted. We hope that we satisfyingly addressed all the comments and that the manuscript will be now suited for publication

Best Regards,

On behalf of all authors

Natalia Vargas, MD

Instituto Nacional de Salud (INS)

Lima, Peru

September 21^st^ 2022

AUTHOR´S RESPONSE TO COMMENTS:

Reviewer #1:

- *Figure 2 legend is still missing a definition of whiskers, bars, etc.*

**RE:** Thank you for your comment, we have added the definitions in the legend and we have enhanced the Figure 2 resolution.

- *Given the differences in immunogenicity between the different vaccines, I would still consider a version of Table 3 separated by the primary vaccination regimen informative (see previous comment on “Lines 243-245/Table 3”). Such a table could go into the supplement.*

**RE:** Thank you for your comment, we have added a separated table as suggested in the supplement section (Supplementary table 3).

- *Lines 351-351 (and previous comment on “Lines 349-351”): While the authors have modified their statement in response to my previous comment, I still consider it misleading. The cited work by Goel has determined that the post-boost “fold-change” in neutralization titer (but not the titer itself) inversely correlated with pre-boost titers. Because the comparison of pre- and post-boost titers in this study is strongly influence by the differences in the cohorts (in terms of baseline vaccine and their typical response to mRNA vaccines – see previous comment), I would suggest removing this paragraph.*

**RE:** Thank you for your comment, we have removed this sentence as suggested.

- *Lines 84-85: The addition of the information on the time between doses is appreciated. However, it would be placed better in the results section when the two cohorts described. Moreover, differentiation by the primary vaccine type (BBIBP-CorV or BNT162b2) would be informative.*

**RE:** Thank you for your comment, we have moved this information to the results section in lines 199 – 200 and also added the time between doses differentiated between the heterologous and homologous vaccine regimens in lines 206 - 208.

- *Line 365: This statement could be worded a bit more careful (“may be” or “can be” “explained” rather than “is” explained).*

**RE:** Thank you for your comment, we have modified the word “is” to “may be” in line 365 as suggested

- *Line 385: Using the term “best” for the description of the used adjustment strategies should be avoided or explained (why are the methods used the ‘best’)?*

**RE:** Thank you for your comment, we have replaced the term “best” by the term “robust” in line 385.
